# Bacterial-based systems for expression and purification of recombinant Lassa virus proteins of immunological relevance

**DOI:** 10.1186/1743-422X-5-74

**Published:** 2008-06-06

**Authors:** Luis M Branco, Alex Matschiner, Joseph N Fair, Augustine Goba, Darryl B Sampey, Philip J Ferro, Kathleen A Cashman, Randal J Schoepp, Robert B Tesh, Daniel G Bausch, Robert F Garry, Mary C Guttieri

**Affiliations:** 1 BioFactura, Inc., Rockville, Maryland, USA; 2 Tulane University Health Sciences Center, New Orleans, Louisiana, USA; 3 Tulane University School of Public Health & Tropical Medicine, New Orleans, Louisiana, USA; 4 Virology Division, United States Army Medical Research Institute of Infectious Diseases, Fort Detrick, Maryland, USA; 5 Diagnostic Systems Division, United States Army Medical Research Institute of Infectious Diseases, Ft. Detrick, Maryland, USA; 6 Lassa Fever Laboratory – Kenema Government Hospital, Kenema, Sierra Leone; 7 University of Texas Medical Branch, Department of Pathology, Galveston, Texas, USA

## Abstract

**Background:**

There is a significant requirement for the development and acquisition of reagents that will facilitate effective diagnosis, treatment, and prevention of Lassa fever. In this regard, recombinant Lassa virus (LASV) proteins may serve as valuable tools in diverse antiviral applications. Bacterial-based systems were engineered for expression and purification of recombinant LASV nucleoprotein (NP), glycoprotein 1 (GP1), and glycoprotein 2 (GP2).

**Results:**

Full-length NP and the ectodomains of GP1 and GP2 were generated as maltose-binding protein (MBP) fusions in the Rosetta strains of *Escherichia coli *(*E. coli*) using pMAL-c2x vectors. Average fusion protein yields per liter of culture for MBP-NP, MBP-GP1, and MBP-GP2 were 10 mg, 9 mg, and 9 mg, respectively. Each protein was captured from cell lysates using amylose resin, cleaved with Factor Xa, and purified using size-exclusion chromatography (SEC). Fermentation cultures resulted in average yields per liter of 1.6 mg, 1.5 mg, and 0.7 mg of purified NP, GP1 and GP2, respectively. LASV-specific antibodies in human convalescent sera specifically detected each of the purified recombinant LASV proteins, highlighting their utility in diagnostic applications. In addition, mouse hyperimmune ascitic fluids (MHAF) against a panel of Old and New World arenaviruses demonstrated selective cross reactivity with LASV proteins in Western blot and enzyme-linked immunosorbent assay (ELISA).

**Conclusion:**

These results demonstrate the potential for developing broadly reactive immunological assays that employ all three arenaviral proteins individually and in combination.

## Background

LASV, a member of the *Arenaviridae *family, is the etiologic agent of Lassa fever, which is an acute and often fatal illness endemic to West Africa. There are an estimated 300,000 – 500,000 cases of Lassa fever each year [1-3], with a mortality rate of 15%–20% for hospitalized patients and as high as 50% during epidemics [4,5]. Presently, there is no licensed vaccine or immunotherapy available for preventing or treating this disease. Although the antiviral drug ribavirin is somewhat beneficial, it must be administered at an early stage of infection to successfully alter disease outcome, thereby limiting its utility [6]. Furthermore, there is no commercially available Lassa fever diagnostic assay, thus preventing early detection and rapid implementation of existing treatment regimens (e.g. ribavirin administration). The lack of adequate countermeasures and means of detection, coupled with the severity of disease, contributed to the classification of LASV as a National Institutes of Allergy and Infectious Diseases (NIAID) Category A pathogen and biosafety level-4 (BSL-4) agent.

The LASV genome is comprised of two ambisense, single-stranded RNA molecules, designated small (S) and large (L) [7]. Two genes on the S segment encode NP, GP1, and GP2; whereas, the L segment encodes the viral polymerase (L protein) and RING finger Z matrix protein. GP1 and GP2 subunits result from post-translational cleavage of a precursor glycoprotein (GPC) by the protease SKI-1/S1P [8]. GP1 serves a putative role in receptor binding, while the structure of GP2 is consistent with viral transmembrane fusion proteins [9].

Humoral immunity to LASV is commonly bipartite, displaying an initial IgM response after infection, with an ensuing mature IgG response [10]. Most diagnostic tests for LASV are currently immunoassay-based and require high containment BSL-4 facilities, using live virus as the source of capture antigen [10]. Such methods are not conducive to field diagnosis, and BSL-4 facilities are not available in areas of the world where LASV is endemic. Thus, it is necessary to develop highly sensitive, reliable, simple, and cost-effective diagnostic assays that can be readily deployed, implemented, and performed in resource-poor settings. Toward this end, we report on the expression, purification, and characterization of LASV proteins in bacterial cell-based systems. Data from these studies clearly demonstrated that the bacterial cell-generated recombinant LASV proteins were immunologically reactive against a panel of suspected LASV convalescent human sera from Sierra Leone and a panel of MHAF against various Old and New World arenaviruses. Collectively, these results demonstrated the putative broad application of these proteins in the diagnosis of arenaviral infections using a narrow range of viral class-specific reagents.

## Results

### Expression and purification of *E. coli*-generated LASV proteins

**Figure 1:**
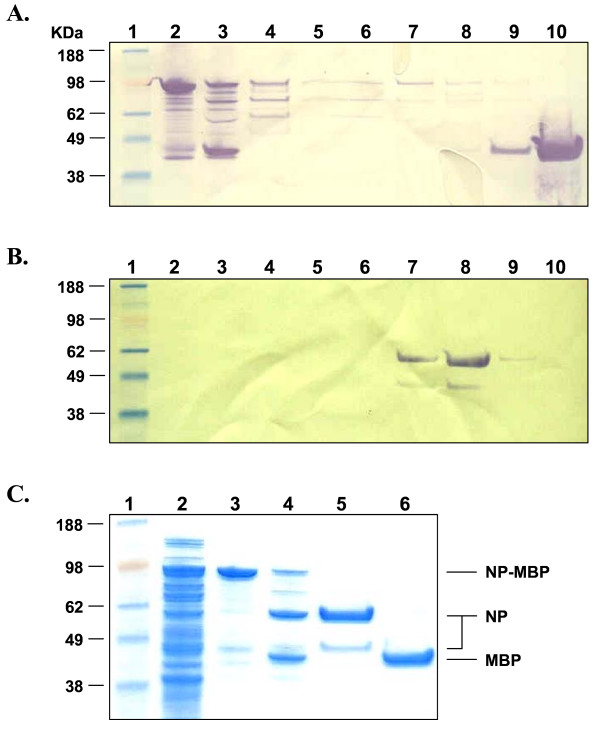
**Expression and purification of LASV NP from *E. coli *Rosetta 2(DE3) cells transformed with construct pMAL-c2x:NP**. An *E. coli *lysate was generated from IPTG-induced cells, the clarified supernatant was applied to an amylose resin column, the protein was eluted with 10 mM maltose, cleaved with Factor Xa, and purified by SEC. (A) Western blot of protein in (lane 2) amylose capture eluate, (lane 3) Factor Xa cleavage reaction, and (lanes 4–10) SEC fractions 4–10. The blot was probed with a rabbit α-MBP polyclonal antibody and then detected with an HRP-conjugated goat α-rabbit IgG antibody. (B) The Western blot in panel A was stripped, reprobed with LASV mAb mix containing NP-specific mAbs, and then detected with an HRP-conjugated goat α-mouse IgG antibody. The identity of each lane is the same as that indicated in Panel A. (C) SDS-PAGE and Coomassie blue stain of proteins in (lane 2) whole bacterial cell lysate, (lane 3) amylose capture eluate, (lane 4) Factor Xa cleavage reaction, (lane 5) SEC-purfied NP generated from pooled NP-containing fractions, and (lane 6) SEC-purified MBP. (Lane 1) SeeBlue^® ^Plus2 pre-stained molecular weight markers, with sizes (kDa) shown to the left of each panel. NP, MBP, and NP-MBP are indicated.

**Figure 2:**
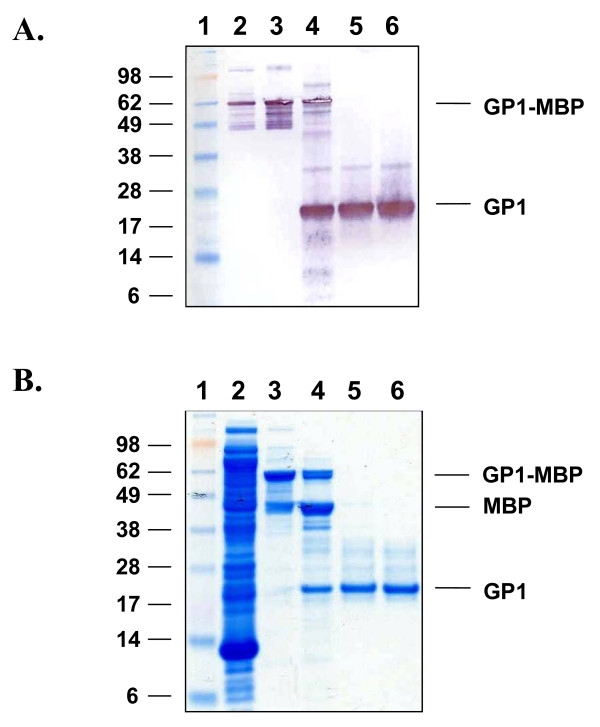
**Expression and purification of LASV GP1 from *E. coli *Rosetta gami 2 cells transformed with construct pMAL-c2x:GP1**. An *E. coli *lysate was generated from IPTG-induced cells, the clarified supernatant was applied to an amylose resin column, the protein was eluted with 10 mM maltose, cleaved with Factor Xa, and purified by SEC. (A) Western blot of protein in (lane 2) whole bacterial cell lysate, (lane 3) amylose capture eluate, (lane 4) Factor Xa cleavage reaction, (lanes 5 and 6) SEC-purified GP1 generated from pooled GP1-containing fractions. The blot was probed with LASV mAb mix containing GP1-specific mAbs, then detected with an HRP-conjugated goat α-mouse IgG antibody. (Lane 1) Western blot XP standard molecular weight markers, with sizes (kDa) shown to the left of the panel. (B) SDS-PAGE and Coomassie blue stain of proteins in (lane 2) whole bacterial cell lysate, (lane 3) amylose capture eluate, (lane 4) Factor Xa cleavage reaction, and (lanes 5 and 6) purified GP1 generated from two sequential SEC runs. (Lane 1) SeeBlue^® ^Plus2 pre-stained molecular weight markers, with sizes (kDa) shown to the left of the panel. GP1, MBP, and GP1-MBP are indicated.

**Figure 3:**
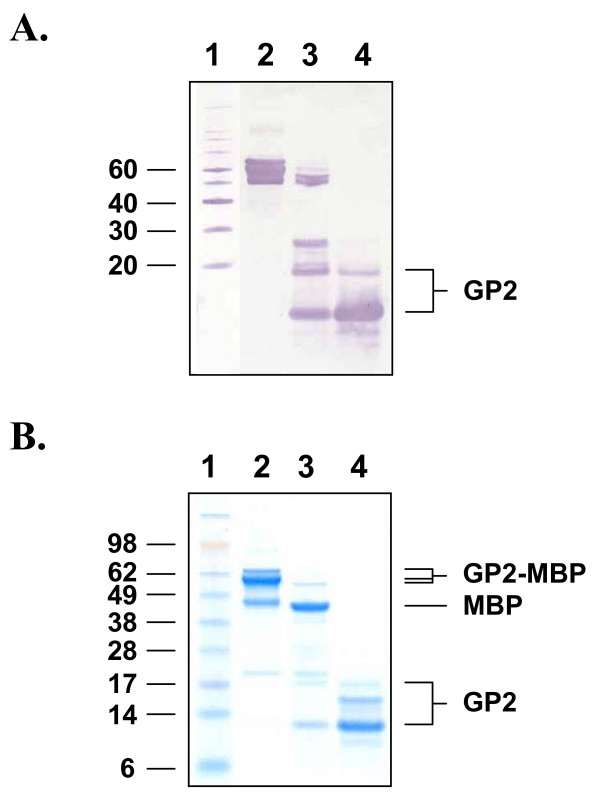
**Expression and purification of LASV GP2 from *E. coli *Rosetta gami 2 cells transformed with construct pMAL-c2x:GP2**. An *E. coli *lysate was generated from IPTG-induced cells, the clarified supernatant was applied to an amylose resin column, the protein was eluted with 10 mM maltose, cleaved with Factor Xa, and purified by SEC. (A) Western blot of protein in (lane 2) amylose capture eluate, (lane 3) Factor Xa cleavage reaction, and (lane 4) pooled SEC fractions. The blot was probed with LASV mAb mix containing GP2-specific mAbs, then detected with an HRP-conjugated goat α-mouse IgG antibody. (Lane 1) Western blot XP molecular weight markers, with sizes (kDa) shown to the left of the panel. (B) SDS-PAGE and Coomassie blue stain of proteins in (lane 2) amylose capture eluate, (lane 3) Factor Xa cleavage reaction, and (lane 4) SEC-purified GP2 generated from pooled GP2-containing fractions. (Lane 1) SeeBlue^® ^Plus2 pre-stained molecular weight markers, with sizes (kDa) shown to the left of the panel. GP2, MBP, and GP2-MBP are indicated.

Expression of full-length LASV NP protein was achieved in *E. coli *Rosetta 2(DE3) cells transformed with vector pMAL-c2x:NP (Figure 1). The ectodomains of LASV GP1 and GP2 proteins were produced in *E. coli *gami 2 cells transformed with vectors pMAL-c2x:GP1 and pMAL-c2x:GP2, respectively (Figures 2 and 3). Specifically, ~98-, 63-, and 65-kDa proteins were detected for MBP-NP, -GP1-, and GP2-fusion proteins, respectively, following isopropyl-β-D-1-thiogalactopyranoside (IPTG) induction (Figures 1, 2, 3). These molecular weights corresponded to the 43-kDa MBP domain fused to the 55-, 22-, and 20-kDa domains of LASV NP, GP1, and GP2, respectively. Western blot analyses revealed that NP and GP1 were primarily expressed as full-length fusion proteins; whereas, expression of MBP-GP2 resulted in a number of truncated forms of the protein (Figures 1, 2, 3). Factor Xa cleavage of the MBP-NP fusion protein resulted primarily in the 55-kDa full-lenth protein and a minor fragment of ~46 kDa in size, as detected by Western blot and sodium dodecyl sulfate polyacrylamide gel electrophoresis (SDS-PAGE) after SEC purification (Figure 1B, lanes 7–8 and 1C, lane 5). Similarly, Factor Xa cleavage of the MBP-GP1 fusion protein resulted primarily in the 22-kDa full-length protein and a minor larger fragment of ca 35-kDa in size, as detected by Western blot (Figure 2A, lanes 4–6). Cleavage of the MBP-GP2 fusion protein and subsequent purification produced two major forms of GP2, a 20-kDa full-length protein and a truncated 13-kDa fragment (Figure 3A, lane 4).

Pilot experiments were performed to determine parameters for optimal fermentation, including criteria for appropriate growth temperature, IPTG concentration, time of harvest following induction, and *E. coli *strain. For optimal expression of MBP-NP fusion protein, pMAL-c2x:NP-transformed Rosetta 2(DE3) cells were induced with 0.03 mM IPTG at 30°C for 4 hours (h). These conditions resulted in an average protein yield of ~12 mg of MBP-NP fusion protein per liter of shake flask culture grown in complete Luria-Bertani Broth (cLB). Initial studies of MBP-GP1 suggested that optimal expression would be achieved with vector pMAL-c2x vector and *E. coli *Rosetta gami 2 cells induced with 0.15 mM IPTG at 22°C for 4 h. However, these conditions ultimately resulted in an average protein yield of only ~0.1 mg of MBP-GP1 fusion protein per liter of culture grown in cLB in shake flasks. Thus, to obtain a sufficient concentration of MBP-GP1 for our studies, it was necessary to generate a cell paste from a 10-L high-density fermentation culture using semi-defined medium and controlled growth parameters, with induction performed at A_600 _= 10. These conditions produced 308 g of cell paste from which ~40 mg of MBP-GP1 fusion protein was isolated. For MBP-GP2, vector pMAL-c2x and *E. coli *Rosetta gami 2 cells were also best suited for expression, with optimal induction performed using 0.15 mM IPTG at 30°C for 4 h. In this manner, an average protein yield of ~13 mg of MBP-GP2 fusion protein was obtained per liter of shake flask culture propagated in cLB. Modifications to growth parameters did not significantly reduce the production of truncated NP or GP2 proteins, pointing to a possible metabolic deficiency in the growth medium or a transcriptional/translational mechanism shortfall.

### Full length and truncated recombinant LASV proteins share predicted N-termini

As identified by SDS-PAGE and Western blot, the major forms of each recombinant LASV protein were sequenced by Edman degradation after cleavage with Factor Xa and purification. Table 1 summarizes the results of N-terminal sequencing for the major bands of each LASV protein. The full length 55-kDa and truncated 46-kDa fragments of LASV NP have identical N-termini, indicating that truncation occurs at a site approximately 9-kDa short of the C-terminus. Similarly, the full length 20-kDa and truncated 13-kDa fragments of LASV GP2 have identical N-termini. LASV GP1 was expressed and purified largely as a single, full length polypeptide with a correctly predicted N-terminus. Thus, recombinant LASV proteins are expressed in these systems with the correct N-termini, and in the case of NP and GP2, the two major truncated forms fall short of reaching the C-terminus during translation in *E. coli *cells.

**Table 1:** N-terminal sequencing of LASV proteins expressed in *E. Coli*

**LASV Protein**	**Protein Form**	**N-terminal sequence**
NP	Full length – 55 kDa	*ISEF ***SASKEI**
NP	Truncated – 46 kDa	*ISEF ***SASKEI**
GP2	Full length – 20 kDa	*ISEFGS ***GTFT**
GP2	Truncated – 13 kDa	*ISEFGS ***GTFT**
GP1	Full Length – 22 kDa	*ISEFGS***TSLYK**

The N-terminus of each recombinant LASV protein cleaved with Factor Xa contains four extraneous amino acids for NP and six for GP1 and GP2 prior to the start of the arenaviral protein sequence. The extraneous amino acids are in italics and the arenaviral protein sequences are in bold.

### Purified recombinant LASV proteins are antigenically recognized by monoclonal antibodies (mAbs) produced against native LASV

**Figure 4:**
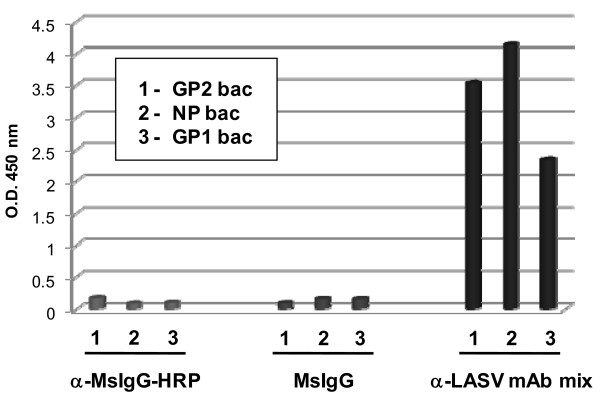
**ELISA of purified recombinant LASV proteins using an α-LASV mAb mix**. ELISA was performed with 100 ng of purified *E. coli*-expressed (1) GP2 (GP2 bac), (2) NP (NP bac), and (3) GP1 (GP1 bac). Proteins were incubated with LASV mAb mix, then detected with an HRP-conjugated goat α-mouse IgG antibody and TMB. For negative controls, proteins were incubated with irrelevant mouse IgG (MsIgG) or with an HRP-conjugated goat α-mouse IgG antibody, then detected as above.

LASV GP1, GP2, and NP proteins generated and purified from *E. coli *were detected by ELISA using a combination of mAbs designated LASV mAb mix, which was comprised of antibodies specific for LASV NP, GP1, and GP2 (Figure 4). Our results were equivalent to those obtained by Western blot analysis of the corresponding denatured proteins (Figures. 1B, 2A, 3A). Collectively, these data suggested that most or all of the epitopes targeted by antibodies in LASV mAb mix are linear. Because this antibody mixture was developed and optimized as a diagnostic reagent for detection of native LASV in clinical samples, there is rationale to suspect that shared linear epitopes in our bacterial-expressed LASV proteins and native viral counterparts may serve as optimal targets for the development of diagnostic immunoassays.

### Purified recombinant LASV proteins are immunologically reactive against LASV-specific convalescent human sera and MHAF against Old and New World arenaviruses

**Figure 5:**
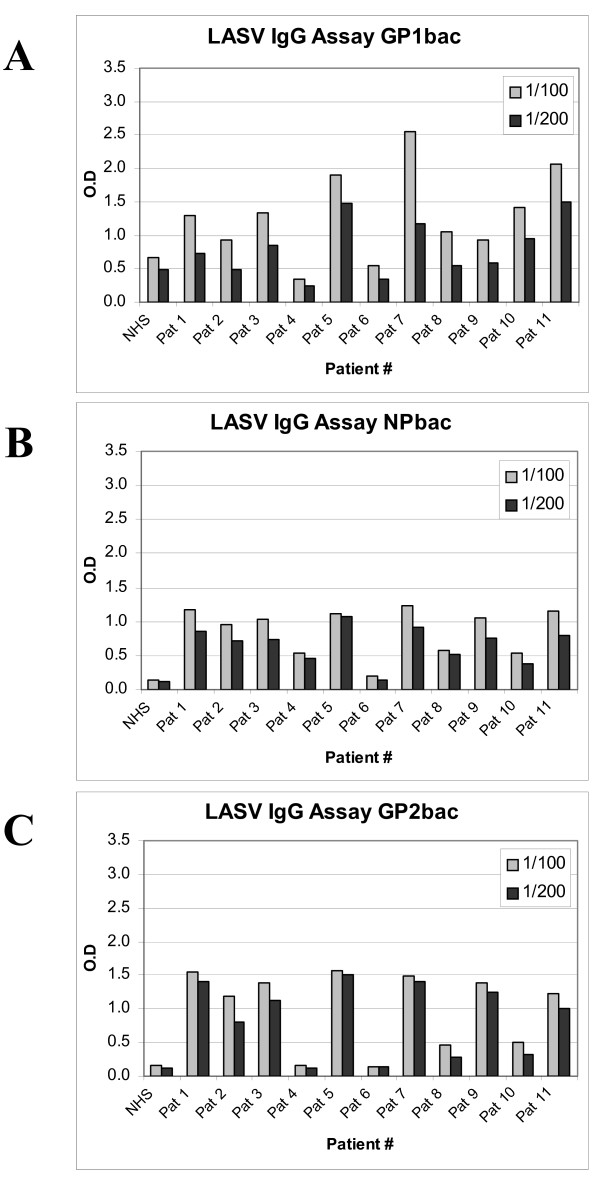
**ELISA of purified recombinant LASV proteins using LASV-specific human convalescent serum**. ELISA was performed with 200 ng of purified *E. coli*-expressed (A) GP1 (GP1 bac), (B) GP2 (GP2 bac), and (C) NP (NP bac). Proteins were incubated with 1:100 (light gray) or 1:200 (dark gray) dilutions of human convalescent serum collected from patients suspected of having previously had Lassa fever or, as a negative control, normal human serum (NHS). Detection was performed with an HRP-conjugated goat α-human IgG antibody and TMB.

As implied above, one of the putative future applications for the LASV proteins generated by these studies is the development of sensitive ELISA-based immunoassays for early detection of Lassa fever in infected patients. Toward this end, we collected human convalescent sera from volunteers suspected of previously having had Lassa fever (no less than 3 months before collection) and, subsequently, assessed the ability of the sera to detect our bacterial cell-generated LASV proteins by ELISA. Here, we report on findings from our initial studies, which were performed using 100- and 200-fold dilutions of 11 serum samples. Purified bacterial-expressed GP1 was detected with statistical significance in 9 of the 11 samples using a 100-fold dilution of sera but only in 7 samples at the higher dilution (Figure 5A). A similar assay detected purified bacterial-expressed NP in 10 of the 11 samples, again with both dilutions (Figure 5B). Purified bacterial-expressed GP2 was detected by ELISA in 9 of 11 samples, with both serum dilutions (Figure 5C). Patient 4 serum specifically detected LASV NP but failed to detect LASV GP1 and GP2. This result may indicate either a Lassa fever-negative outcome or a potential IgM-positive response, without detectable IgG class switch. Thus, these preliminary data may support a growing body of evidence, which suggest that the humoral immune response to LASV infection is biased towards LASV NP [11-13]. If proven true, NP may be the most relevant immunological marker for early detection of Lassa fever; whereas, a detectable immune response to GP1 and GP2 antigens may follow a more mature humoral response to infection. We could not detect any of the bacterial-expressed LASV proteins with patient 6 serum, which may also reflect either a Lassa fever-negative outcome or an IgM-mediated response to infection.

LASV GP1 generated the lowest signal-to-noise ratio of the 3 bacterial-expressed proteins tested. In patient samples 1, 2, 8, and 9, statistically significant detection of LASV GP1 was attained using a 100-fold dilution of sera but not with a 200-fold dilution (Figure 5A). This twofold dilution resulted in a significant decrease in the specific detection of GP1, with an average decline of 37.5% per sample; whereas, the average % decline in detection for ELISA of GP2 and NP was 17.7 and 23.6, respectively. This observation may reflect a lower concentration of GP1-specific antibodies, lower affinity specificities, or simply a lower representation of antibodies directed to non-native epitopes represented in the bacterial-expressed antigen. None of the recombinant LASV proteins were specifically detected by sera from Lassa fever naïve donors (Figure 5, lane "NHS"), resulting in the acquisition of data that were statistically comparable to those obtained with all seronegative patient samples.

To further investigate the utility of our recombinant LASV proteins for functional applications, we used Western blot and ELISA to test 4 Old and 5 New World arenavirus-specific MHAFs for their ability to cross-react with bacterial-expressed LASV NP, GP1, and GP2 (Table 2). The MHAFs were generated against unprocessed arenavirus-infected murine brain extracts and thus contained native viral proteins, which could have elicited a murine immune response targeted against linear and conformational epitopes. Purified LASV NP cross-reacted significantly with most MHAFs of Old and New World origin, with the exception of Latino virus-specific MHAF. LASV GP2 was the second most cross-reactive protein to heterologous MHAFs. In addition, there was a close correlation between the cross-reactivity observed for NP and that of GP2. With the exception of lymphocytic choriomeningitis (LCMV)-and Pirital-specific MHAF, which reacted weakly to NP and did not react with GP2, and Latino virus-specific MHAF, which did not react with either, all other MHAF showed dual reactivity. Bacterial-expressed GP1 bound only to Mobala, Mopeia, and Pichinde virus-specific MHAF and thus exhibited the least cross-reactivity against the panel tested. Collectively, most of the MHAFs yielded ELISA data similar to the results obtained by Western blot analysis. The most pronounced differences were observed when comparing binding data of MHAFs to GP1 protein. Only Mobala- and Pichinde-specific MHAF bound to GP1 by Western blot, and when tested by ELISA, only Mobala-specific MHAF exhibited significant binding to the protein, with Mopeia- and Tamiami-specific MHAFs reacting to a lesser extent.

**Table 2:** Cross reactivity of Old and New World arenavirus-specific MHAFs against recombinant LASV GP1, GP2, and NP proteins by Western blot and ELISA

		NP bac		GP1 bac		GP2 bac	
MHAF	Distribution	WB	ELISA	WB	ELISA	WB	ELISA

NMS	-	-	-	-	-	-	-

LCMV	Old World	+/-	-	-	-	-	-
Ippy	Old World	++	+/-	-	-	++	+++
Mobala	Old World	++	++	+	+	++	++
Mopeia	Old World	++	++	-	+/-	+	++

Latino	New World	-	-	-	-	-	-
Tamiami	New World	+/-	-	-	+/-	+/-	+/-
Pirital	New World	+	+/-	-	-	-	-
Pichinde	New World	+	-	++	-	+	+/-
Oliveros	New World	+/-	-	-	-	+/-	-

Western blot (WB) and ELISA were performed with 100 ng of purified *E. coli*-expressed NP (NP bac), GP1 (GP1 bac), and GP2 (GP2 bac). WB abbreviations: (-) negative, no visible band detected; (+/-) faint band detected; (+) bright band detected; (++) very bright band detected. ELISA abbreviations (all signals are respective to corrected background): (-) negative; (+/-) < 2X; (+) > 2X < 3X; (++) > 3X < 4X; (+++) > 4X < 5X.

## Discussion

LASV proteins were produced in bacterial cell lines using the MBP fusion-based pMAL-vector system (New England BioLabs, Ipswich, MA), comprised of pMAL-p2x and -c2x bacterial expression vectors. The former plasmid utilizes a periplasmic signal that translocates recombinant proteins to the periplasmic space of *E. coli*; whereas, the latter vector contains a mutation in the translocation signal and thus will yield only cytoplasm-associated recombinant proteins. Selection of vector pMAL-c2x for expression of LASV NP, GP1, and GP proteins was determined by two critical observations we made during our small-scale pilot experiments: (1) the -p2x vector background generated significantly less recombinant protein per gram of cell mass than the -c2x counterpart, an observation that has been extensively documented in the literature and in the manufacturer's manual for the pMAL expression system (pMAL Protein Fusion and Purification System Manual, New England BioLabs); and (2) translocation of LASV GP1 and NP to the periplasmic space of *E. coli *was toxic to the host cells (data not shown). Although we demonstrated that all 3 LASV proteins could be translocated to and purified from the periplasmic space, NP- and GP1-containing cells either yielded no fusion protein or lysed upon centrifugation and/or osmotic shock. Thus, to develop reproducible and scalable protein production and purification processes, we investigated LASV protein expression in the intracellular space using vector pMAL-c2x. This approach, however, was met with another potential obstacle, as the intracellular space of *E. coli *is a reducing environment and is, therefore, not conducive to expression of proteins that require disulfide bond formation for correct folding. This represented a critical point for consideration with regard to GP1 and GP2, which are believed to contain secondary structures formed by disulfide bond-mediated constraining, as per current proposed models [9]. For our studies, we therefore expressed the glycoproteins in the *E. coli *Rosetta gami 2 strain, which contains mutations in the *trxB *and *gor *genes and thus permits disulfide bond formation in the cytoplasm. Ultimately, the combination of an *E. coli *Rosetta gami 2 strain and the pMAL-c2x vector background resulted in improved expression of both LASV glycoproteins, allowing us to achieve the highest yield of recombinant protein per gram of cell mass in an environment appropriate for generation of conformationally correct protein. LASV NP expression also benefited from the use of vector pMAL-c2x; however, as this protein is not thought to possess secondary structures that are influenced by a reducing environment, the *E. coli *Rosetta 2(DE3) strain was used rather than gami 2 cells. Although higher concentrations of NP per unit of cell mass were achieved with pMAL-c2x when compared to the -p2x counterpart, a significant portion of the protein was contained in insoluble fractions after cell lysis. Recently, Sletta *et al. *[14] demonstrated the critical role served by prokaryotic translocation signal sequences in achieving industrial-level expression of proteins with medical relevance for humans. Thus, expression technologies that exploit secretory mechanisms may alleviate difficulties encountered with proteins such as LASV NP, which aggregate as insoluble matter in the cytoplasm and are cytotoxic when translocated to the periplasmic space of the cell. We are therefore interested in identifying expression elements that facilitate improved expression of all 3 LASV proteins in *E. coli*, while maximizing protein integrity and yield in a manner that permits production of higher concentrations of full-length product. Furthermore, we are currently exploring alternative purification schemes to alleviate difficulties we encountered with the Factor Xa cleavage system, which was expensive and often resulted in non-specific uncoupling of fusion domains.

Although bacterial-expressed full-length LASV proteins were produced, we also obtained truncated versions of the proteins to varying degrees. We repeatedly co-eluted a minor 46-kDa protein along with full-length 55-kDa NP (Figure 1). The truncated form of NP was equally detected by the 2 LASV NP-specific mAbs contained in LASV mAb mix, as determined by Western blot (Figure 1B). Expression and purification of LASV GP2 primarily yielded a truncated 13-kDa fragment and a full-length 20-kDa protein (Figure 3). At least 2 other minor fragments, which were each less than 13-kDa in size, were also detected in most preparations. The observed ratio of 13-versus 20-kDa proteins obtained in the final pooled GP2-containing fractions appeared to reflect the expression profile in the *E. coli *environment rather than an artefact of the purification scheme, as deduced by our analyses. We repeatedly detected four GP2 protein bands by Coomassie staining and SDS-PAGE of the amylose capture eluate from IPTG-induced Rosetta gami 2:pMAL-p2x-(data not shown) and -c2x MBP-GP2-containing cell extracts (Figure 3B). Three bands, 50-, 55-, and 65-kDa in size, corresponded to various forms of GP2, with the largest band representing the full length fusion protein, as determined on Western blots detected with LASV mAb mix (Figure 3A). Conversely, the fourth protein represented MBP, as it was detected by Western blot analysis using MBP-specific antisera (data not shown) but not LASV mAb mix. Collectively, these data suggested potential arrest points in the expression of the LASV glycoproteins in this prokaryotic system, which may have resulted from a transcriptional or translational impairment that allowed for production of the full-length protein in addition to truncated species. Our methodology did not permit us to determine if metabolic proteolysis during recombinant protein synthesis was the source of truncated protein production. In addition, we were unable to determine if the fermentation process contributed to these results, as minimal medium optimization was performed. Conversely, expression of LASV GP1 resulted primarily in production of the full-length 22-kDa protein, which was detected on Western blots by LASV mAb mix (Figure 2A). The fermentation parameters we used to produce GP1 employed an enriched medium to sustain high-density *E. coli *propagation, which resulted in improved volumetric yields of full-length GP1 when compared to the yield obtained from low-density shake flask cultures. Future improvements to this system(s) will be required to generate higher levels of full-length LASV proteins for diagnostic and potential therapeutic applications. Initial development efforts will concentrate on improving volumetric yields of each LASV protein using optimized fermentation parameters and enriched media aimed at reducing the metabolic burden associated with high level expression of eukaryotic viral proteins in *E. coli*.

As our intention is to use the recombinant proteins we generated for development of an ELISA-based diagnostic assay, we conducted several immunological studies by which we demonstrated the ability of our bacterial cell-expressed proteins to bind to LASV-specific mAbs and human sera, as well as arenavirus-specific MHAF. Our results clearly suggested the practical use of the bacterial-expressed proteins for this purpose. Although full characterization and comparison of bacterial-versus mammalian-generated LASV proteins will be required to identify broadly shared epitopes in each relevant protein by all available and future antibody reagents, current data support the development of bacterial-expression platforms, which are cost effective and thus a desired avenue for protein production. However, it will be necessary to establish that post-translational modifications, such as the predicted 7 N-linked glycosylation sites in LASV GP1 and 4 in GP2, are not critical for broad antigen detection by native human antibodies in infected patient sera. Although Lassa fever convalescent serum IgGs may recognize linear and conformational epitopes in the bacterial-expressed glycoproteins, an additional immunoglobulin fraction may be directed against native epitopes, which may include glycosylated domains. These comparative studies will be facilitated through the generation and extensive characterization of panels of mAbs to native (mammalian-expressed) and non-native (bacterial-expressed) LASV proteins.

A compilation of results from Western blot, ELISA, or both using MHAF against Old and New World arenaviruses inferred the potential for developing broadly reactive immunological assays that employ all three LASV proteins concurrently. This is reflected by the data in Table 2, which indicated that each of the bacterial-expressed LASV proteins effectively detected antibodies in MHAFs specific for Old and New World arenaviruses. Bowen *et al*. [15] reported un-rooted phylogenetic trees for LASV NP, GP1, and GP2, showing relationships among arenaviruses. Alignment of NP sequences indicated that LASV strains Josiah, GA39, 803213, and Ip are all more closely related to Mopeia than any strain of the prototype arenavirus LCMV. Also, Pichinde and Oliveros were more distantly related to LASV strains than Mopeia and LCMV. Overall, our results revealed disparities between statistically calculated relatedness among arenavirus strains of multiple origins and corresponding immunological cross-reactivities to recombinant LASV proteins with MHAFs. For example, reactivity of Pichinde MHAF to LASV GP1 would not have been expected based on the observed lack of binding by more closely related arenavirus MHAFs, such as Ippy and LCMV. Data suggested that differences among relevant arenaviral protein sequences may account for variation in epitope immuno-dominances. Highly conserved epitopes in NP and the glycoproteins among arenaviruses may not result in similar humoral responses upon viral exposure, thus yielding polyclonal antibody pools that are biased toward more immuno-dominant, yet more diverse sequences. Conversely, if highly conserved epitopes in the proteins of more distantly related arenaviruses are more immuno-dominant than more heterogeneous sequences, the resulting humoral response may result in detectable cross-reactivity across arenaviral classes and subtypes. Although confirming this supposition would require fine epitope mapping, it could explain the lack of reactivity by MHAFs against arenaviruses closely related to LASV, while exhibiting strong binding to more distant counterparts.

## Conclusion

Collectively, this work provides a gateway for development of a recombinant protein ELISA-based system for early diagnostic detection of arenaviral infections in human subjects using sera samples collected in the field. Toward this end, subsequent work will be aimed at generating a broad panel of mAbs against all of the LASV proteins described in these studies. These antibodies will be used as both capture and detection reagents in the production of sensitive diagnostic immunoassays to, not only LASV, but to other arenaviruses as well. Additional studies will be performed to characterize these mAbs *in vitro *and to explore their potential protective efficacy using *in vivo *animal models. Thus, these studies could result in a panel of reagents that will greatly improve diagnosis of Lassa fever in endemic regions of the world. The classification of Lassa fever and other arenaviruses by the U.S Government as Category A agents with Biowarfare potential further justifies the development of countermeasures against this highly virulent class of viruses.

## Methods

### Virus, cells, plasmids, antibodies, human sera, and MHAF

LASV, strain Josiah [16], was propagated in Vero cells (ATCC CRL 1587), which were maintained in complete Eagle's Minimal Essential medium (cEMEM) containing non-essential amino acids (NEAA) supplemented with 10% heat-inactivated fetal bovine serum (ΔFBS) and 20 μg/mL of gentamicin. All plasmid constructs were engineered in *E. coli *strain DH5α, according to the manufacturer's instructions (Invitrogen, Carlsbad, CA). LASV proteins were expressed in *E. coli *Rosetta 2(DE3) and gami 2 strains (Novagen, Madison, WI), which contain the chloramphenicol-resistant plasmid pRARE, encoding tRNAs for six (pRARE1) or seven (pRARE2) rare codons (AUA, AGG, AGA, CUA, CCC, GGA, and CGG) aimed at enhancing expression of eukaryotic proteins in prokaryotic systems. Rosetta gami 2 cells contain *trxB *and *gor *mutations, which permit disulfide bond formation in the cytoplasm. Large-scale shaker flask cultures of *E. coli *Rosetta strains expressing LASV NP and GP2 were performed in cLB medium supplemented with 2 g/L of glucose, 100 μg/mL of ampicillin, and 35 μg/mL of chloramphenicol. Large-scale fermentation of the *E. coli *Rosetta strain expressing LASV GP1 was performed in semi-defined batch medium comprised of 40 g/L yeast extract, 4.0 g/L potassium phosphate monobasic (KH_2_PO_4_), 11.33 g/L sodium phosphate dibasic heptahydate (Na_2_HPO_4_), 6.0 g/L ammonium sulfate ((NH_4_)_2_SO_4_), 0.2 g/L of uridine, 2 g/L of glucose, 0.372 mL/L of Dawes Trace 1, 2.14 mL/L of Dawes Trace 2, 0.072 mL/L of Dawes Trace 3, 0.606 mL/L of 1 M calcium chloride dihydrate (CaCl_2_-2H_2_O), 0.30 mL/L of 0.43 g/mL thiamine-HCl, 333 μL/L of 30% (v/v) Antifoam A (Sigma), 35 mg/L of chloramphenicol, and 100 mg/L of carbenicillin.

The MBP fusion-based pMAL-vector system (New England BioLabs), comprised of pMAL-p2x and -c2x vectors, was used for production of LASV proteins. Both plasmids contain protease recognition sites that permit Factor Xa cleavage of recombinant proteins from MBP after purification. We analyzed LASV protein sequences for the presence of the Factor Xa cleavage recognition sequence (IQGR) before choosing this protease for our studies. No sites were found that were identical to this sequence or to published non-specific cleavage sequence sites [17,18].

For immunoassays, Dr. Randal Schoepp kindly provided the following LASV-specific mAbs: NP-specific mAbs 52-273-8 and L2-54-6A; GP1-specific mAb L52-74-7A; and GP2-specific mAbs L52-272-7, L52-121-22, and L52-272-7, which were produced against purified gamma-irradiated LASV, as previously described [19]. These mAbs were used individually, in various combinations, or in a mixture designated LASV mAb mix that was comprised of all the mAbs. Preliminary work indicated that LASV mAb mix was well suited for detecting native and denatured LASV proteins, respectively (data not shown). Rabbit anti-MBP polyclonal antibody was purchased from New England BioLabs. Horseradish peroxidase (HRP)-conjugated secondary antibodies specific for mouse and rabbit IgG were purchased from Kirkegaard and Perry Laboratories (KPL, Gaithersburg, MD).

Human convalescent sera were collected from healthy volunteers suspected to have previously had Lassa fever, as determined by retrospective differential diagnosis from patient records at the Kenema Government Hospital (Kenema, Eastern District, Sierra Leone) in accordance with the National Institutes of Health's DMID Protocol Number 06–0008. Blood samples were not obtained from individuals whom had been sick within 3 months prior to collection in order to insure that any previous Lassa infection would be resolved. Each patient was given informed consent prior to donating blood. Briefly, whole blood was collected from volunteers in 5 mL serum Vacutainer^® ^tubes, (Becton Dickinson Biosciences, San Jose, CA) and allowed to clot for 1 h at 4°C. Serum was decanted into cryogenic tubes and labelled with unique numerical patient identifiers. As an additional precautionary measure, the samples were heat-inactivated for 1 h at 60°C, which has been shown to completely inactivate LASV, and then stored at -20°C until transported to the United States. Serum samples were shipped at ambient temperature in licensed storage containers using a commercial courier, according to International Air Transport Authority (IATA) and U.S. government regulations regarding the shipment of diagnostic specimens. Upon receipt, 0.025% (w/v) sodium azide was added to each tube and samples were stored at -20°C until further use.

Specific MHAF were prepared against each of the following arenaviruses at the World Reference Center for Emerging Viruses and Arboviruses, University of Texas Medical Branch (UTMB): LCMV, Ippy, Mobala, Mopeia, Latino, Tamiami, Pirital, Pichinde, and Oliveros viruses. Briefly, the immunogens were 10% (w/v) crude brain homogenates of infected mouse brain in phosphate-buffered saline (PBS). The vaccination schedule consisted of four weekly injections of mouse brain antigen mixed with Freund's adjuvant. After the fourth injection, sarcoma 180 cells were injected intraperitoneally in mice to induce ascites formation. The ascitic fluid was removed by paracentesis when the abdomen became distended. MHAF production was done under a UTMB-approved animal protocol. Normal mouse serum (NMS) was used as a negative control in Western blots and ELISA.

### LASV propagation, cDNA synthesis, and polymerase chain reaction (PCR) amplification of LASV genes

Vero cells were infected with LASV strain Josiah at a multiplicity of infection of 0.1. Briefly, virus was diluted in cEMEM to a final volume of 2.0 mL, then added to confluent cells in a T-75 flask and incubated for 1 h at 37°C, with 5% CO_2 _and periodic rocking. Subsequently, 13 mL of cEMEM was added, and the culture was incubated in a similar manner for 96 h. To prepare total cellular RNA, the cell culture medium was replaced with 2 mL of TRIzol™ reagent (Invitrogen), and total RNA was purified according to the manufacturer's specifications. Using the ProtoScript First Strand cDNA Synthesis Kit (New England BioLabs), 100 ng of total cellular RNA per reaction was transcribed into cDNA, as outlined in the manufacturer's protocol. The Phusion™ High-Fidelity PCR Mastermix (New England BioLabs) was used in all amplifications of LASV gene sequences. PCR parameters were determined based on the melting temperature for each oligonucleotide set. LASV GP1 and GP2 genes were amplified using the following cycling conditions: 98°C for one 15 second (sec) cycle and then 35 repeated cycles of 98°C for 5 sec, 59°C for 10 sec, and 72°C for 15 sec, followed by a final extension at 72°C for 5 minutes (min). LASV NP was amplified using the following cycling conditions: 98°C for one 30 sec cycle and then 35 repeated cycles of 98°C for 10 sec, 59°C for 15 sec, and 72°C for 30 sec, followed by a final extension at 72°C for 5 min.

Table 3 outlines each of the nucleotide sequences of the oligonucleotide primers used in the amplification of LASV genes for expression in bacterial cell systems. The ectodomain of the LASV GP1 gene, lacking a signal sequence and the N-terminal methionine (N-Met), was amplified using (1) a 41-mer forward oligonucleotide primer (5' GP1 bac), which contained a *Bam *HI restriction endonuclease (REN) site and comprised the N-terminal 8 amino acids (a.a.) of the mature GP1 protein beyond the known SPase cleavage site; and (2) a 49-mer reverse oligonucleotide primer (3' GP1 bac), which contained a *Hind *III REN site, as well as two termination codons, and comprised the C-terminal 10 a.a. of the mature GP1 protein. The ectodomain of the LASV GP2 gene was amplified using (1) a 38-mer forward oligonucleotide primer (5' GP2 bac), which contained a *Bam *HI REN site and comprised the N-terminal 7 a.a. of the mature GP2 protein beyond the known SKI-1/S1P protease cleavage site; and (2) a 40-mer reverse oligonucleotide primer (3' GP2 bac), which contained a *Hind *III REN site, as well as two termination codons, and comprised the C-terminal 7 a.a. of the GP2 protein preceding the start of the native transmembrane (TM) anchor domain. The LASV NP gene sequence was amplified using (1) a 77-mer forward oligonucleotide primer (5' NP bac), which contained an *Eco *RI REN site and comprised the N-terminal 22 a.a. of the polypeptide without the N-Met; and (2) a 43-mer reverse oligonucleotide primer (3' NP bac), which contained a *Hind *III REN site, as well as two termination codons, and comprised the C-terminal 8 a.a. of the NP protein.

**Table 3:** Oligonucleotide primers used for amplification of LASV genes expressed in E. coli

**LASV Gene Amplified**	**LASV Primer**	**Oligonucleotide Primer Sequence**
GP1	5' GP1 bac	TTTCAGAATTCGGATCCACCAGTCTTTATAAAGGGGTTTAT
GP1	3' GP1 bac	GGTACCAAGCTT**TCA**G**TCA**TAGCAATCTTCTACTAATATAAATATCTCT
GP2	5' GP2 bac	TTTCAGAATTCGGATCCGGCACATTCACATGGACACTG
GP2	3' GP2 bac	GGTACCAAGCTT**TCA**G**CTA**TGTCTTCCCCTGCCTCTCCAT
NP	5' NP bac	TTTCAGAATTCAGTGCCTCAAAGGAAATAAAATCCTTTTTGTGGACACAATCTTTGAGGAGGGAATTATCTGGTTAC
NP	3' NP bac	GGTACCAAGCTT**TCA**G**TTA**CAGAACGACTCTAGGTGTCGATGT

*Note*. REN sites are underlined, and stop codons (TCA, CTA, TTA) are in bold print.

### Cloning LASV genes for expression in bacterial cell systems

**Figure 6:**
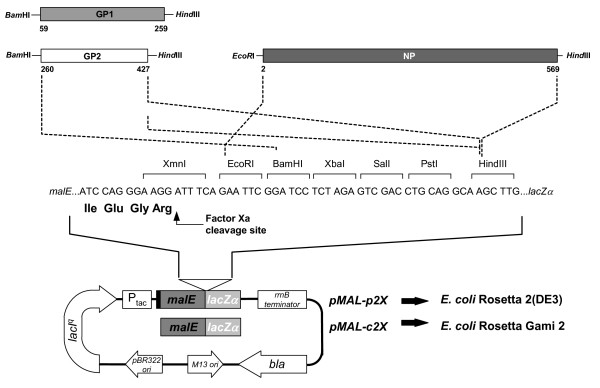
**Cloning strategy for expression of LASV proteins GP1, GP2, and NP in *E. coli *using pMAL vectors**. To generate MBP-LASV gene fusions for *E. coli *expression, PCR-amplified LASV gene sequences were restricted and cloned in-frame at the 3' end of the *mal*E gene, beyond the cleavage site for Factor Xa (IQGR). The LASV GP1 gene sequence comprised a.a. 59–259 in the native GPC, spanning the first a.a. beyond the known SPase cleavage site at position 58 to the junction between GP1 and GP2 domains, which is cleaved by the SKI-1/S1P protease at a.a. 259. The LASV GP2 gene sequence comprised a.a. 260–427, spanning the first a.a. of mature GP2 to the last a.a. before the predicted TM domain. The LASV NP gene sequence comprised the complete ORF of the gene, with the exception of the N-terminal Met. The 3' oligonucleotides used for amplification of each gene sequence were engineered to contain two terminator codons separated by a single nucleotide. All genes were cloned into vectors pMAL-p2x and pMAL-c2x for periplasmic and cytoplasmic expression of fusion proteins, respectively, in *E. coli *Rosetta 2(DE3) or gami 2 strains. The a.a. position of each LASV gene domain is noted, as are REN sites. Abbreviations include: MBP gene (*mal*E), MBP promoter (P_tac_), philamentous phage origin of replication (M13 ori), bacterial origin of replication (pBR322 ori), beta-lactamase gene (*bla*), *E. coli *terminator (rrnB), the LacZ alpha-complementation domain (LacZα), and the lacI repressor gene (lacI^q^). The periplasmic secretory domain in pMAL-p2x is indicated by a black box on the 5' end of the *mal*E gene sequence.

Figure 6 summarizes the strategy used to clone LASV GP1, GP2, and NP gene sequences into vectors pMAL-p2x and -c2x for expression in bacteria. The constructs and *E. coli *strains used to express the recombinant LASV genes are outlined in Table 4. Briefly, initial pilot expression studies were performed with vectors pMAL-p2x:GP1, pMAL-p2x:GP2, and pMAL-p2x:NP in the Rosetta 2(DE3) *E. coli *strain. Subsequent experiments used vectors pMAL-c2x:GP1, pMAL-c2x:GP2, and pMAL-c2x:NP, with the former two constructs expressed in *E. coli *Rosetta gami 2 cells and the latter in *E. coli *Rosetta 2(DE3) cells. DNA was manipulated by standard techniques [20], and all recombinant plasmids outlined in Table 4 were initially engineered and propagated in *E. coli *DH5α.

**Table 4:** Summary of vectors and respective *E. coli *strains used to express recombinant LASV genes

**Recombinant Plasmid**	**LASV Gene**	**Expression System**
pMAL-p2x:GP1	GP1	Rosetta 2(DE3)
pMAL-p2x:GP2	GP2	Rosetta 2(DE3)
pMAL-p2x:NP	NP	Rosetta 2(DE3)
pMAL-c2x:GP1	GP1	Rosetta gami 2
pMAL-c2x:GP2	GP2	Rosetta gami 2
pMAL-c2x:NP	NP	Rosetta 2(DE3)

### Optimization of recombinant LASV protein expression in bacteria

Small-scale pilot experiments were performed with each pMAL-p2x or -c2x construct to determine optimal bacterial expression conditions for each MBP-LASV fusion protein. Briefly, 50-mL shaker flask cultures of transformed *E. coli *were grown in cLB at 22°C, 30°C, and 37°C to an A_600 _= 0.5–0.6. Each culture was split into three flasks and induced with IPTG to final concentrations of 0.03, 0.15, and 0.3 mM. Cultures were then grown under induction conditions for 2 h. Subsequently, periplasmic and cytoplasmic fractions were prepared by osmotic shock of *E. coli *transformed with pMAL-p2x-based vectors and by generation of whole cell lysates of *E. coli *transformed with pMAL-c2x-based vectors, respectively. MBP-LASV fusion proteins were captured from each fraction on amylose resin (New England BioLabs) and then analyzed by SDS-PAGE under reducing conditions. Using optimal temperature and IPTG parameters determined by the above studies, a time-course investigation was carried out to further maximize total fusion protein yields. SDS-PAGE analysis was performed on LASV-MBP fusion proteins captured on amylose resin from samples harvested at 2, 3, and 4 h after induction.

### Scheme for small-scale purification of recombinant LASV proteins expressed in bacteria

LASV-MBP fusion proteins were purified from whole cell lysates of *E. coli *transformed with pMAL-c2X-based vectors by capture on amylose resin followed by Factor Xa cleavage, according to the manufacturer's instructions (New England BioLabs). The addition of dithiothreitol (DTT) was necessary to prevent aggregation and precipitation of protein before and during Factor Xa cleavage of LASV GP1-MBP and GP2-MBP fusion proteins. Moreover, the addition 0.03 to 0.05% SDS was required for efficient Factor Xa cleavage of both these fusion proteins. Briefly, cleaved LASV proteins were separated from MBP and other contaminants using a Superdex 200 Prep Grade size-exclusion column (Amersham Biosciences, Pittsburgh, PA). To prevent aggregation, 30 mM 2-(N-morpholino)ethanesulphonic acid (MES) buffer containing 0.1% (w/v) SDS was required for SEC of Factor Xa-treated GP2-MBP fusion protein. SEC of Factor Xa-treated GP1-MBP fusion protein required 30 mM MES buffer containing 5 mM DTT and 0.1% (w/v) SDS. LASV NP-MBP was cleaved with Factor Xa alone and was purified by SEC using 1× PBS, pH 7.4. These conditions were subsequently applied to the large-scale purification schemes of the respective LASV proteins.

### Large-scale production and purification of recombinant LASV proteins expressed in bacteria

#### LASV NP

To generate and purify LASV NP, a 3-L shaker flask culture of pMAL-c2x:NP-transformed Rosetta 2(DE3) cells was grown in cLB to an A_600 _= 0.5–0.6 at 30°C and then induced with a final IPTG concentration of 0.03 mM. After incubation at 30°C for 4 h, cells were harvested by centrifugation for 10 min at ~13,000 × *g*. The cell paste was frozen at -20°C and subsequently thawed and resuspended in 9 volumes of lysis buffer (20 mM TrisHCl, 200 mM NaCl, 10 mM EDTA, pH 8.0). Next, a bacterial protease inhibitor cocktail (Sigma) and lysozyme (Pierce Biotechnology, Rockford, IL) were added at concentrations of 1 mL per 4 grams and 40 mg per gram of wet cell paste, respectively, and the suspension was incubated at 37°C, with agitation. After 30 min, 1/10 volume of 1 M MgSO_4 _and 50 μL of 2000 U/mL DNase I (Roche, Nutley, NJ) per gram wet cell paste were added. The solution was incubated for an additional 30 min at 37°C and then centrifuged at 13,000 × *g *for 60 min at 4°C. The resulting supernatant was further clarified by 0.2-μm filtration, then diluted two-fold with lysis buffer and applied to a 1.6 × 10 cm amylose column at 75 cm/h. The column was washed with 5 column volumes of equilibration buffer (20 mM TrisHCl, 200 mM NaCl, pH 7.4), and the fusion protein was eluted with equilibration buffer containing 10 mM maltose. For every A_280 _= 1 of fusion protein, 20 μL of 1 mg/mL Factor Xa (Novagen) was added. The reaction mixture was then incubated overnight at 4°C and subsequently clarified by low-speed centrifugation followed by 0.2-μm filtration. The solution was loaded onto a 2.6 × 70 cm Superdex 200 Prep Grade size-exclusion column (Amersham Biosciences) in 6-mL aliquots and eluted at 60 cm/h with 1× PBS, pH 7.4. LASV NP-containing fractions were pooled and concentrated using an Amicon stirred cell unit fitted with a 10,000 NMWL ultrafiltration membrane (Millipore, Billerica, MA) at 20 psig Nitrogen. Purified LASV NP was sterile filtered using a 0.2-μm Millex GV syringe filter (Millipore), then distributed to vials and stored at -20°C.

#### LASV GP1

To express and purify LASV GP1, a 10-L culture of pMAL-c2x:GP1-transformed Rosetta gami 2 cells was grown in semi-defined batch medium at 37°C using a New Brunswick Scientific fermentor (Edison, NJ). When the density of the culture reached A_600 _= 7.75, a yeast extract feed consisting of 200 g/L yeast extract, 0.33 g/L uridine, 0.3 g/L histidine, and 0.3 g/L methionine was started at a rate of 4 mL/min. When the density of the culture reached A_600 _= 10.0, the temperature was reduced to 22°C, the yeast extract feed was reduced to 2 mL/min, and IPTG was added to a final concentration of 3.0 mM. During the fermentation, dissolved oxygen was set at 40%, and the culture was supplemented with 50% glucose to maintain its concentration between 0.2–2 g/L. At 4 h post-induction (A_600 _= 13.7), cells were harvested by centrifugation for 10 min at ~13,000 × *g*. The resulting cell paste was frozen at -80°C and subsequently thawed and resuspended in 9 volumes of lysis buffer (20 mM TrisHCl, 200 mM NaCl, 10 mM EDTA, 1 mM DTT, pH 8.0). As described above for NP purification, bacterial protease inhibitor cocktail and lysozyme were added to the suspension, and the reaction was incubated at 37°C, with agitation. After 45 min, 1/10 volume of 1 M MgSO_4 _and 50 μL of 2000 U/mL DNase I (Roche) per gram wet cell paste were added. The solution was incubated for an additional 30 min at 37°C and then centrifuged at 10,000 × *g *for 60 min at 0°C. The supernatant was further clarified by 0.2-μm filtration and processed in two cycles as follows: the supernatant was applied to a 2.6 × 8.0 cm amylose column at 75 cm/hr, the column was washed with 5 column volumes of equilibration buffer (20 mM TrisHCl, 200 mM NaCl, 1 mM EDTA, 1 mM DTT, pH 7.4), and the fusion protein was eluted with equilibration buffer containing 10 mM maltose. SDS and EDTA were added to a final concentration of 0.05% (w/v) and 2 mM, respectively, followed by the addition of 5 μL of 1 mg/mL Factor Xa (Novagen) per A_280 _= 1 of amylose column eluate. After overnight incubation at 2–8°C, DTT was added to a final concentration of 5 mM and the solution was concentrated five-fold using an Amicon stirred cell fitted with a 4,000 NMWL PLBC ultrafiltration membrane (Millipore) at 40 psig at room temperature. Subsequently, the solution was 0.2-μm-filtered and loaded onto a 2.6 × 70 cm Superdex 200 Prep Grade size-exclusion column (Amersham Biosciences) in 6 mL aliquots and eluted with 30 mM MES, 154 mM NaCl, 0.1% SDS, 5 mM DTT, pH 6.7, at 30 cm/h. The fractions containing full-length GP1 were pooled and then concentrated as before to ~12 mL. To remove high-molecular-weight contaminants and DTT, LASV GP1 was re-run on the Superdex 200 column with 30 mM MES, 154 mM NaCl, 0.1% SDS, pH 6.7. The GP1 eluate pool was stored overnight at 2–8°C to precipitate SDS. Precipitated SDS was removed from the concentrated sample by centrifugation at 10,000 × *g *at 0°C for 1 h. Purified LASV GP1 was immediately sterile filtered using a 0.2-μm Millex GV syringe filter (Millipore), then distributed to vials and stored at -20°C.

#### LASV GP2

To express and purify LASV GP2, a 3-L shaker flask culture of pMAL-c2x:GP2-transformed Rosetta 2(DE3) cells was grown at 30°C to an A_600 _= 0.5–0.6 in cLB and then induced with a final IPTG concentration of 0.15 mM. After incubation for 3.5 h at 30°C, the cells were harvested by centrifugation for 10 min at ~13,000 × *g*. The resulting cell paste was frozen at -20°C, then thawed and resuspended in 9 volumes of lysis buffer (20 mM TrisHCl, 200 mM NaCl, 10 mM EDTA, 1 mM DTT, pH 8.0). As described above, bacterial protease inhibitor cocktail and lysozyme were added to the suspension, and the reaction was incubated at 37°C, with agitation. After 30 min, 1/10 volume of 1 M MgSO_4 _and 50 μL of 2000 U/mL DNase I (Roche) per gram wet cell paste were added. The solution was incubated for an additional 30 min at 37°C and then centrifuged at 15,000 × *g *for 15 min at 4°C. The supernatant was further clarified by 0.2-μm filtration, then diluted twofold with lysis buffer and applied to a 1.6 × 11 cm amylose column at 75 cm/h. The column was washed with 5 column volumes of equilibration buffer (20 mM TrisHCl, 200 mM NaCl, 1 mM EDTA, 1 mM DTT, pH 7.4), and the fusion protein was eluted with equilibration buffer containing 10 mM maltose. SDS was added to a final concentration of 0.03% (w/v), followed by 10 μL of 1 mg/mL of Factor Xa (New England BioLabs) per A_280 _= 1 of amylose column eluate. After incubation for 17 h at 4°C, the solution was concentrated threefold using an Amicon stirred cell unit fitted with a 3,000 NMWL ultrafiltration membrane (Millipore) at 30 psig Nitrogen. Subsequently, the solution was loaded onto a 2.6 cm × 70 cm Superdex 200 Prep Grade size-exclusion column in ~6 mL-aliquots and then eluted with 30 mM MES, 154 mM NaCl, 0.1% SDS, pH 6.7, at 30 cm/h. GP2-containing fractions were pooled and concentrated, as described for GP1 purification. The sample was further concentrated with a Centriplus YM-3 unit (Millipore) at 2,500 × *g *at room temperature and stored overnight at 4°C. Precipitated SDS was removed by centrifugation at 2,500 × *g *at 0°C. Purified LASV GP2 was immediately sterile filtered using a 0.2 μm Millex GV syringe filter (Millipore), then distributed to vials and stored at -20°C.

### Western blot analysis of recombinant LASV proteins

The identity of LASV proteins generated in bacterial systems was confirmed by Western blot analysis using 10–15 μL of LASV mAb mix at a 1:1,000 dilution or a 1:100 dilution of MHAF. Briefly, proteins were transferred to 0.45-μM nitrocellulose membranes using XCell II™ Blot Modules, according to the manufacturer's instructions (Invitrogen). Blocking and probing of membranes were performed in 1× PBS, pH 7.4, 5% non-fat dry milk, 0.05% Tween-20, and 0.01% thymerosal. Membranes were washed with 1× PBS, pH 7.4, 0.1% Tween-20 (PBST, wash buffer). Detection was performed with 10–15 mL of 1 μg/mL of HRP-conjugated goat α-mouse IgG (H+L) polyclonal antibody reagent (KPL) and tetramethylbenzidine (TMB) membrane substrate. Reactions were stopped by immersing developed membranes in water, followed by immediate high resolution scanning for permanent recording. When applicable, blots were stripped in 62.5 mM Tris-HCl, pH 6.7, 5 mM EDTA, 2% SDS, 100 mM β-mercaptoethanol, for 1 h at 50°C in a sealed plastic bag, with shaking. Stripped membranes were subsequently washed extensively in wash buffer, then blocked and re-probed, as described above.

### ELISA

ELISA was performed with NP, GP1, and GP2 proteins generated in *E. coli*. Briefly, high-affinity Costar 3590 (Costar) or Nunc PolySorp (Nunc) 96-well plates were coated with purified proteins at a final concentration of 0.1 or 0.2 μg per well in PBS, pH 7.5. Plates were incubated overnight at 4°C and washed three times with PBST. Plates were then blocked for 90 min with 200 μL of blocking buffer consisting of 5% milk in PBST, then washed as above. A 1:1,000 dilution of LASV mAb mix, 1:100 or 1:200 dilutions of human convalescent sera, or a 1:100 dilution of MHAF in blocking buffer was added at a final volume of 100 μL/well, and the plates were incubated for 1 h at 37°C, then washed as above. Detection was performed with 100 μL/well of HRP-conjugated goat α-mouse IgG (H+L) polyclonal antibody reagent (KPL) or HRP-conjugated Fc-specific human anti-IgG antibody (Bethyl Laboratories, Montgomery, TX) diluted to 1 μg/mL in blocking buffer. After 1 h incubation, 100 μL/well of TMB substrate (KPL) was added, and the plates were incubated for 5 min. The reaction was stopped by adding 100 μL/well of TMB stop solution (KPL) and read at 450 nm in a Molecular Dynamics ThermoMax spectrophotometer, using SoftMax Pro analysis software (Molecular Devices Corp., Sunnyvale, CA).

### N-terminal protein sequencing

N-terminal sequence determination by Edman degradation was carried out in an Applied Biosystems Model 4949 CLC protein suquenator. Phenylthiohydantoin derivatives of amino acids were analyzed on-line with an Applied Biosystems Model 785A/140C/610A analyzer. All reagents and solvents were from Applied Biosystems.

## Competing interests

Luis M Branco, Alex Matschiner, and Darryl B Sampey are co-founders and sole members of the Board of Directors of BioFactura, Inc., and have received salary and other compensation from the company, such as founder's stock, as it pertains to the execution of this work. This publication may, in part, result in the seeking of additional funding, public or private, to support follow-up studies pertinent to the work outlined herein. Luis M Branco, Alex Matschiner, Darryl B Sampey, Joseph N Fair, Robert F Garry, and Mary C Guttieri are listed inventors, in addition to others, in a PCT application entitled "Soluble and Membrane-Anchored Forms of Lassa Virus Subunit Proteins", filed in April 2008.

## Authors' contributions

LMB contributed to the experimental design, engineered the expression systems, performed data analysis, and drafted the manuscript, AM developed purification methods for each of the proteins, JNF contributed to the *in vitro *analysis of recombinant antibodies and viral proteins, facilitated acquisition of convalescent immune sera, and assisted with development of ELISA formats, AG assisted with collection of convalescent human immune sera, DBS performed large scale fermentation and expression of recombinant viral proteins in bioreactors, PJF provided Trizol™ suspensions prepared from LASV-infected cell cultures, KAC assisted with engineering of bacterial expression systems, RJS provided LASV-specific mAbs, RBT provided MHAFs, DGB provided critical assistance in obtaining convalescent human sera, RFG contributed to the experimental design and provided critical review of the manuscript, MCG contributed to the experimental design, procurement of critical reagents, data analysis, drafting and critical review of the manuscript.
